# Evaluation of the mediastinal-thoracic volume ratio on postmortem computed tomography

**DOI:** 10.1007/s00414-021-02593-0

**Published:** 2021-04-28

**Authors:** Vasiliki Chatzaraki, Lars C. Ebert, Michael J. Thali, Anna-Bettina Haidich, Garyfalia Ampanozi

**Affiliations:** 1 Department of Forensic Medicine and Imaging, Institute of Forensic Medicine, University of Zurich, Zurich, Switzerland; 2 Department of Radiology, Kantonsspital Baden, Baden, Switzerland; 3 Department of Hygiene, Social-Preventive Medicine and Medical Statistics, Medical School, Aristotle University of Thessaloniki, Thessaloniki, Greece

**Keywords:** Cardiothoracic ratio, Three-dimensional (3D), Cause of death, Segmentation, Postmortem computed tomography, Virtopsy

## Abstract

**Objectives:**

The aim of this study was to measure the mediastinal-thoracic volume ratio (CTR_VOL) on PMCT as a more accurate version of traditional CTR, in order to assess the terminal positional relationship between the heart and lungs in the different causes of death with regard to age, gender, BMI, cardiomegaly, and lung expansion.

**Materials:**

Two hundred fifty consecutive postmortem cases with pre-autopsy PMCT and full forensic autopsy were retrospectively evaluated. The lungs and the mediastinum were manually segmented on the PMCT data and the correspondent volumes were estimated in situ. CTR_VOL was calculated as the ratio of the mediastinal to the thoracic volume. The volume measurements were repeated by the same rater for the evaluation of the intrarater reliability. Age, gender, body weight and height, heart weight at autopsy, and cause of death were retrieved from the autopsy reports. Presence of lung expansion was radiologically evaluated in situ.

**Results:**

CTR_VOL was positively associated with age and BMI but not with gender and was higher for cardiomegaly compared to normal hearts, lower for asphyxiation-related deaths compared to cardiac deaths and intoxications, and lower for cases with lung expansion. The intrarater reliability was excellent for the calculated volumes of both lungs and mediastinum.

**Conclusion:**

The results of the present study support CTR_VOL as a tool to assess the relationship between the heart and lungs in situ, which differs significantly between the studied cause of death categories.

**Supplementary Information:**

The online version contains supplementary material available at 10.1007/s00414-021-02593-0.

## Introduction

Cardiothoracic ratio (CTR) is used in clinical radiology for the evaluation of cardiomegaly. It is calculated by dividing the maximum cardiac with the maximum thoracic horizontal diameter and a value equal to 0.5 is defined as threshold for cardiomegaly diagnosis [1]. CTR has been also applied in the postmortem setting [2–4] and a new threshold of 0.57 was suggested for determining cardiomegaly based on postmortem computed tomography (PMCT) [2]. However, the terminal cardiac dilatative changes lead to CTR overestimation and false-positive diagnosis of cardiomegaly [3].

According to Michiue et al. [5], CTR on 367 chest radiographs was larger for cadavers that died because of heart disease/intoxication compared to drowning/asphyxiation and the diaphragm levels were lower in the second group indicating lung hyperinflation [5]. Cardiac hypertrophy, dilatation, and blood volume overload seem to primarily affect the postmortem CTR [5]. In another study, Michiue et al. showed lower cardiac dilatation index in mechanical asphyxia than in sudden cardiac deaths [6]. Sogawa et al. [7, 8] assessed the heart to both lungs’ volume ratio in different causes of death (*n* = 70) and this was higher in sudden cardiac deaths (≈ 0.4) compared to drowning and mechanical asphyxia (both ≈ 0.24) [8]. In intoxications (≈0.3) the ratio was variable and rather close to that of sudden cardiac deaths indicating a combination of pre-existing cardiac enlargement and acute pulmonary atelectasis [8].

Cardiac deaths are typically associated with cardiac cavities’ congestion and cardiogenic pulmonary edema at autopsy [9–11]. Intoxications show non-specific findings with generalized congestion of organs and vessels, brain, and pulmonary edema [9]. On the other hand, all kinds of suffocation (smothering, gagging, choking, chemical asphyxia by air-poison inhalation), mechanical asphyxia (positional asphyxia, traumatic asphyxia), and manual/ligature strangulation can show congestion of the organs, particularly of the right heart, acute pulmonary emphysema, and hemorrhagic edema. In hanging, besides organ congestion, lung hyperinflation may be present or not, depending on what extent the asphyxiation-aspect has taken place. In drowning, lungs are acutely hyperinflated, large, and bulky, almost or completely touching each other in the anterior mediastinal space, so-called emphysema aquosum [9–12]. Hypothermia is histologically associated with pulmonary intra-alveolar edema with interstitial hemorrhages [10]; however, lung hyperinflation has been recently reported [13].

This study intended to evaluate the relationship between mediastinum and lungs on PMCT in a large sample with measurements of the mediastinal-thoracic volume ratio, as a more accurate, three-dimensional (3D) version of the one-dimensional CTR, with regard to different causes of death categories, age, gender, BMI, presence of cardiomegaly and lung hyperinflation.

## Materials and methods

### Subjects

**Fig. 1:**
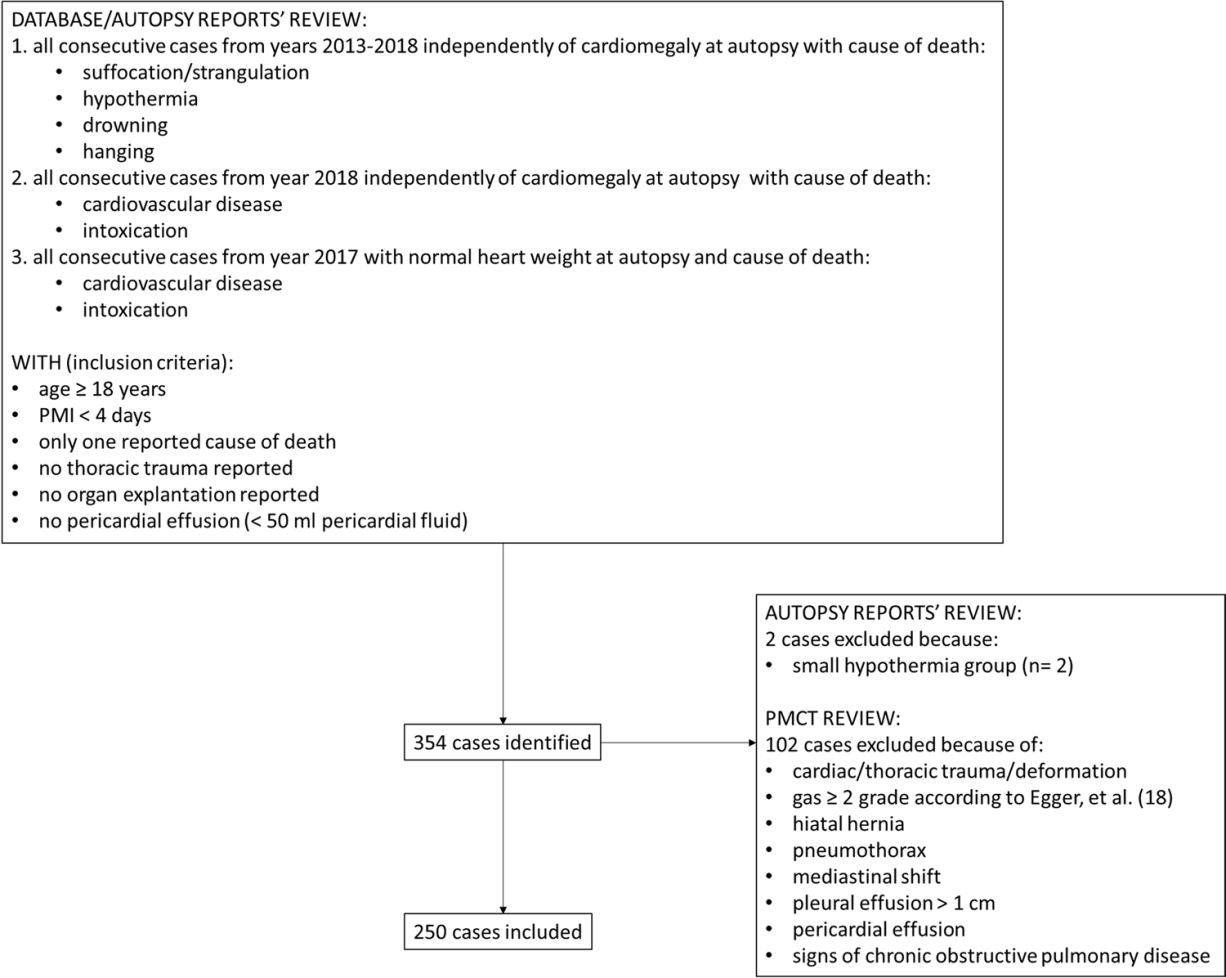
Study flow chart

Autopsy reports of all consecutive cases with pre-autopsy PMCT and full autopsy of the years 2013–2018 were reviewed in our database retrospectively. The reported cause of death and heart weight measured at autopsy for the determination of cardiomegaly based on the method by Zeek [14] were noted. Cardiovascular causes of death and intoxications appear more often than asphyxiation-related deaths in our institute. Additionally, the number of cases with cardiomegaly prominently exceeds those with normal heart at autopsy, which was also observed in previous studies with consecutive case selection [2]. In order to avoid large inequalities among the distinct groups, all consecutive cases with asphyxiation-related death of the years 2013–2018 (independently of cardiomegaly), all consecutive deaths related to cardiovascular causes or intoxication only of the year 2018 (independently of cardiomegaly), and all consecutive cases related to cardiovascular events or intoxication *having normal heart weight* only from the year 2017 were selected (Fig. 1).

Suffocation, strangulation, asphyxiation, drowning, and hanging were considered asphyxiation-related deaths. Hypothermia cases were also included in this group because of hyperinflated lungs on PMCT [13, 15]. Cardiovascular causes included cardiac infarctions, coronary thrombosis, fatal rhythm disturbances, pulmonary thromboembolism, and acute cardiac arrest. Intoxication group included all intoxications independently of substance. Two cause of death categories were further created: “Cardiac-intoxication” category contained the subjects with cardiovascular causes of death or intoxication and “asphyxiation” category contained suffocation, strangulation, hypothermia, drowning, and hanging.

Exclusion criteria related to autopsy reports contained age < 18 years, postmortem interval (PMI) > 4 days based on the estimation of time of death [11, 16, 17], more than one reported causes of death, thoracic trauma, organ explantation, and pericardial effusion (pericardial fluid volume at autopsy > 50 ml).

One observer with 3 years’ experience in forensic imaging assessed PMCT axial images for meeting the following radiological exclusion criteria: moderate/extended gas accumulations in the thoracic organs and vessels as defined by Egger et al. [18], thorax deformation, cardiac trauma, mediastinal shift, pneumothorax, pleural effusions with > 1 cm distance from dorsal pleura, pericardial effusion, hiatal hernia, and any kind of distortion of the pulmonary architecture as sign of chronic obstructive pulmonary disease [19].

Gender, body height, and body weight were retrieved from the autopsy reports and BMI was further calculated for the cases that were finally included.

### Imaging

PMCT was performed on a 128-slice scanner (SOMATOM Definition Flash, Siemens Healthcare, Forchheim, Germany) with the bodies in a supine position, using automatic dose modulation (CARE Dose 4D, Siemens Healthcare, Forchheim, Germany). Imaging parameters were [20] tube voltage 120 kVp and slice collimation 128 × 0.6 mm. PMCT image reconstructions of thorax-abdomen were performed [20] with slice thickness of 5.0 mm.

**Fig. 2:**
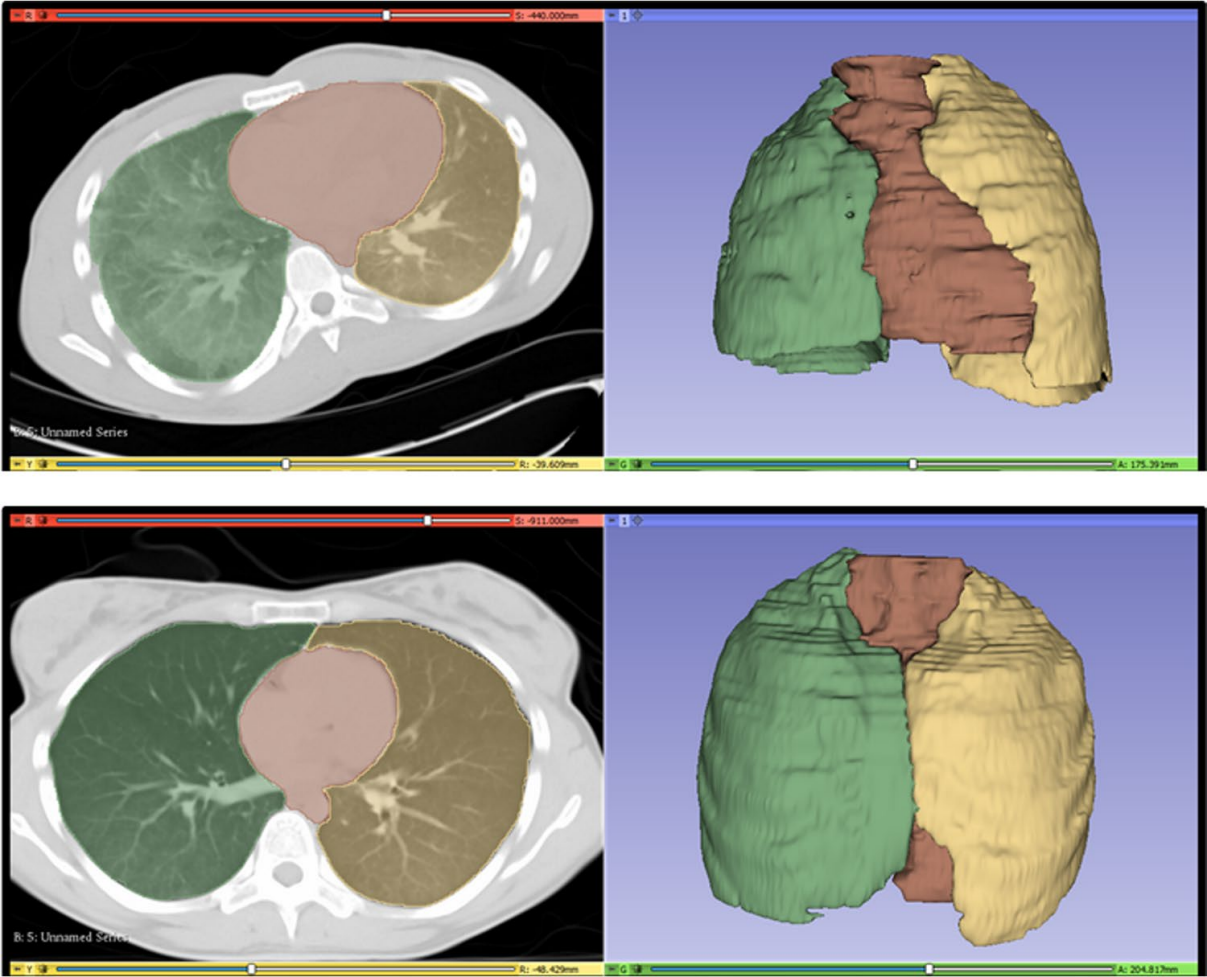
3D Slicer environment presenting the final 3D models of two cases after segmentation of the right lung (green), the left lung (orange). and mediastinum (red). On the top: Case evaluated without lung expansion on PMCT; the lungs do not touch each other at the anterior mediastinal space. Heart weight was normal at autopsy. CTR_VOL was calculated 25.3%. On the bottom: Case evaluated as positive for lung expansion; the lungs touch each other anteriorly. Heart weight was normal at autopsy. CTR_VOL was calculated 14.7%

The same observer noted whether lungs touched each other at the anterior mediastinal space, as a sign of lung hyperinflation on PMCT. Cases were subsequently divided into two groups: no lung expansion and lung expansion group (Fig. 2).

### Segmentation-volume estimation–mediastinal-thoracic volume ratio

**Fig. 3:**
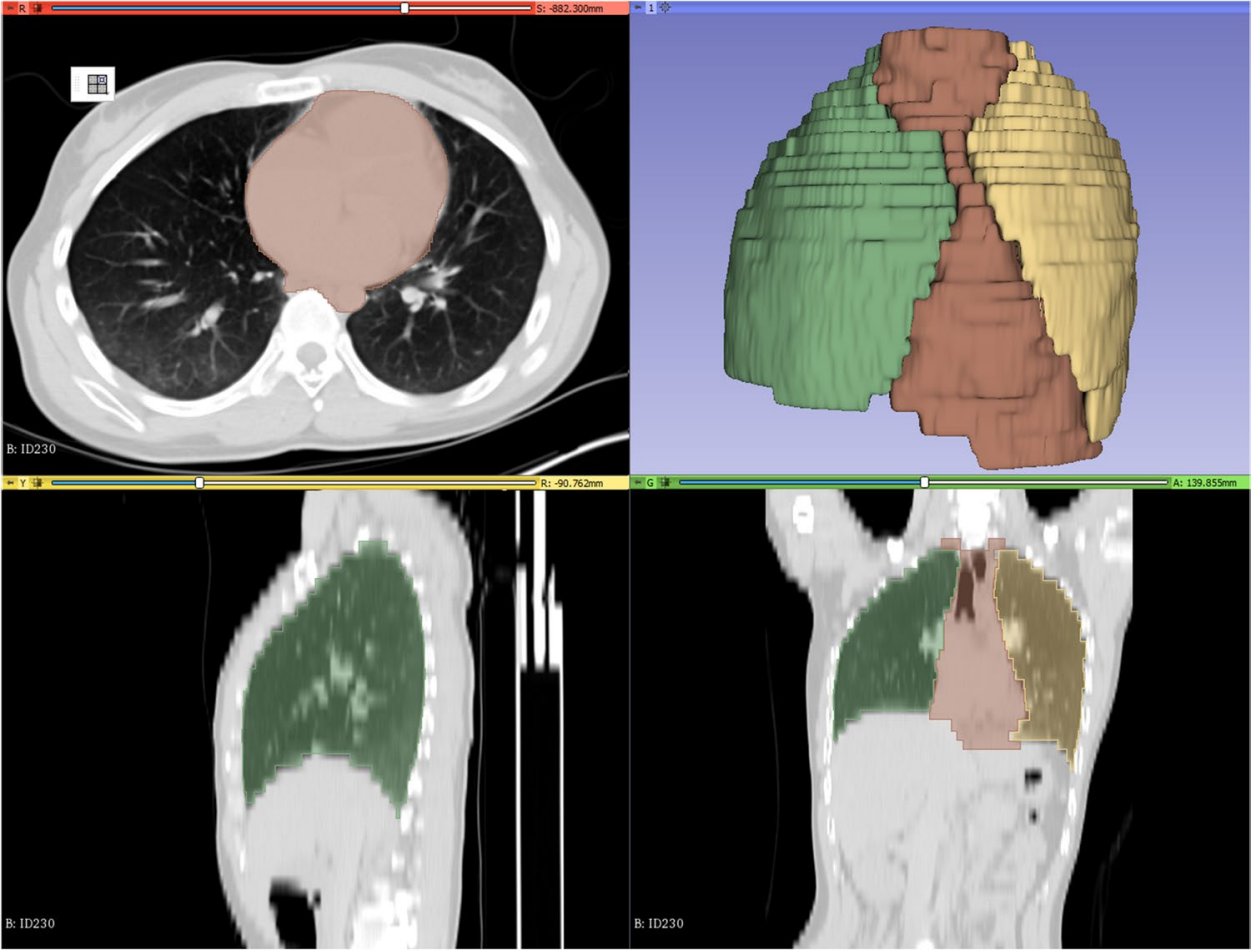
3D Slicer environment with the PMCT data of a male with cause of death hanging, normal heart weight at autopsy, and no lung expansion on PMCT: Segmentation of the right lung (green), the left lung (yellow), the mediastinum (red) with the final 3D model. Hilus, vessels, and whole pulmonary tissue independent of internal livors or edema were included in the pulmonary volume. Paracardial fat tissue was included in the mediastinal volume measurements. CTR_VOL% was calculated 25.6% in this case

PMCT data of the thorax-abdomen in soft tissue kernel and 5 mm thickness were anonymized, given a study-specific identification number, extracted from the database and imported in 3D Slicer 4.10.1 software (open source, free software platform for medical image informatics, image processing, and 3D visualization, http://www.slicer.org [21]). Both lungs (including hilus, vessels, and whole parenchyma independently of internal livors or edema) and mediastinum (superior border at the level of 1^st^ ribs, paracardial fat tissue included) were manually segmented in every axial slice (Fig. 3). In order to speed up the segmentation procedure, the PMCT data were down-sampled to 10 mm in axial orientation according to Ebert et al. [22] (see also Supplementary material).

The mediastinal-thoracic ratio (CTR_VOL) was calculated based on the calculated volumes as follows:

$$\mathrm{CTR}\_\mathrm{VOL}=\frac{\mathrm{mediastinal volume}}{\mathrm{thoracic volume}}=\frac{\mathrm{mediastinal volume}}{\mathrm{right} \mathrm{lung volume}+\mathrm{left lung volume}+\mathrm{mediastinal volume}}$$

### Intrarater agreement

To increase accuracy, the segmentations were repeated for the whole study sample by the same observer in the same manner after an approximately one and a half year period. During the repeat measurements, the case list was randomly reordered, and the observer was blinded to the measurements obtained by the first review.

### Statistical analysis

Shapiro–Wilk tests were performed for checking normality of distribution. Continuous variables were presented as mean and standard deviation (SD). Levene’s test was performed for checking homogeneity of variances, independent samples *t*-test (Student’s for pooled and Welch’s for unpooled variances), and Kruskal–Wallis test (non-parametric ANOVA) with post hoc analysis with Bonferroni correction to determine differences between groups. Linear regression analysis was used to investigate factors associated with CTR_VOL. The multivariable model was built with backward elimination method, where univariate variables with *p*-values lower than 0.20 were considered for inclusion. Intraclass correlation coefficient (ICC) estimates and their 95% confident intervals (CIs) were calculated for assessing the intrarater reliability based on two-way mixed-effects model, single rater type, and absolute agreement for intrarater reliability [23]. Analysis and plots were computed with R (R Core Team (2018). R: A language and environment for statistical computing. R Foundation for Statistical Computing, Vienna, Austria. URL https://www.R-project.org/). All *p*-values were two-tailed at 5% significance level.

## Results

### Subjects

Three hundred fifty-four cases were identified during the autopsy reports’ review. One hundred two cases were excluded after PMCT review. Because the hypothermia group included only 2 cases, these were also excluded considered insufficient for a reliable statistical evaluation. Thus, 250 cases were finally included (Fig. 1, Table 1). From these 250, the majority (68%) concerned males with mean age 48.3 (SD: 18.9) and the rest 81 (32%) females with mean age 53.3 (SD: 19.3). Most subjects had cause of death related to cardiovascular causes (32%), while the other most frequent causes of death were hanging (20%), drowning (19%), and intoxication (18%). Lung expansion was present in one-third of subjects (30%) while the majority had cardiomegaly (61%).

**Table 1:** Descriptive table depicting the baseline characteristics for whole sample (*N* = 250)

Mean age in years (SD)	49.8 (19.1)
Mean BMI in kg/m^2^ (SD)	25.8 (5.6)
Mean right lung volume in L (SD)	1.58 (0.42)
Mean left lung volume in L (SD)	1.37 (0.41)
Mean mediastinal volume in L (SD)	1.22 (0.33)
Mean CTR_VOL (SD)	0.3 (0.07)
Cause of death, *n* (%)	
Cardiac	80 (32)
Intoxication	44 (17.6)
Suffocation-strangulation	29 (11.6)
Drowning	47 (18.8)
Hanging	50 (20)
Lung expansion, *n* (%)	
Yes	75 (30)
No	175 (70)
Cardiomegaly, *n* (%)	
Yes	152 (60.8)
No	98 (39.2)

### Volume calculation–mediastinal-thoracic volume ratio

**Fig. 4:**
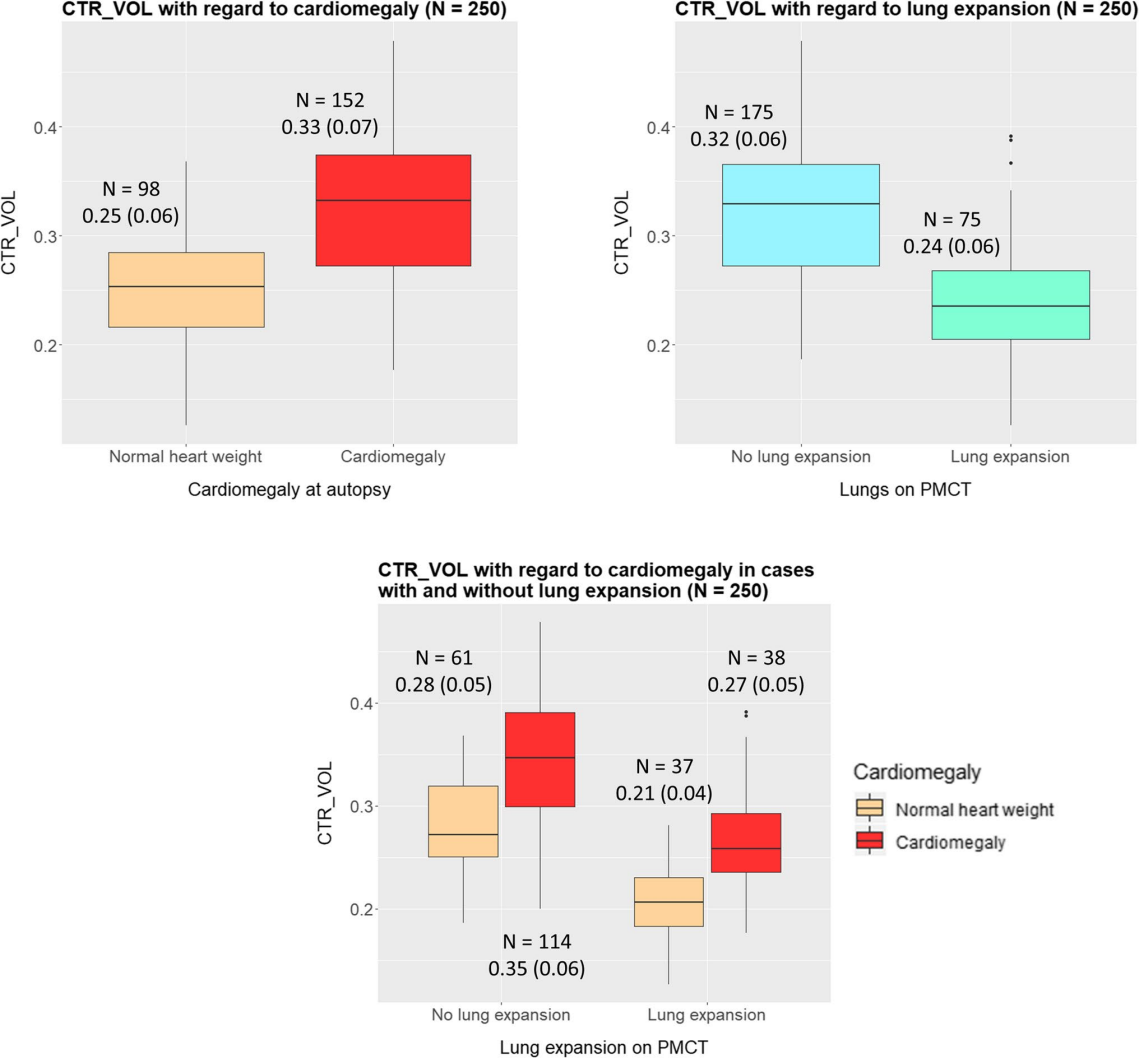
Left on the top: Boxplots depicting CTR_VOL with regard to cardiomegaly. CTR_VOL was greater for cardiomegaly compared to normal hearts (Welch’s *t*-test: *p* < 0.001). Right on the top: CTR_VOL with regard to lung expansion. CTR_VOL was lower in lung expansion compared to no expansion (Welch’s *t*-test: *p* < 0.001). On the bottom: CTR_VOL with regard to cardiomegaly in the distinct lung expansion groups. CTR_VOL was greater for cardiomegaly compared to normal hearts within the distinct lung expansion groups (within both expansion and no expansion: Student’s *t*-test: *p* < 0.001). CTR_VOL was lower in lung expansion compared to no expansion within the distinct heart groups (within both cardiomegaly and normal hearts: Student’s *t*-test: *p* < 0.001)

**Fig. 5:**
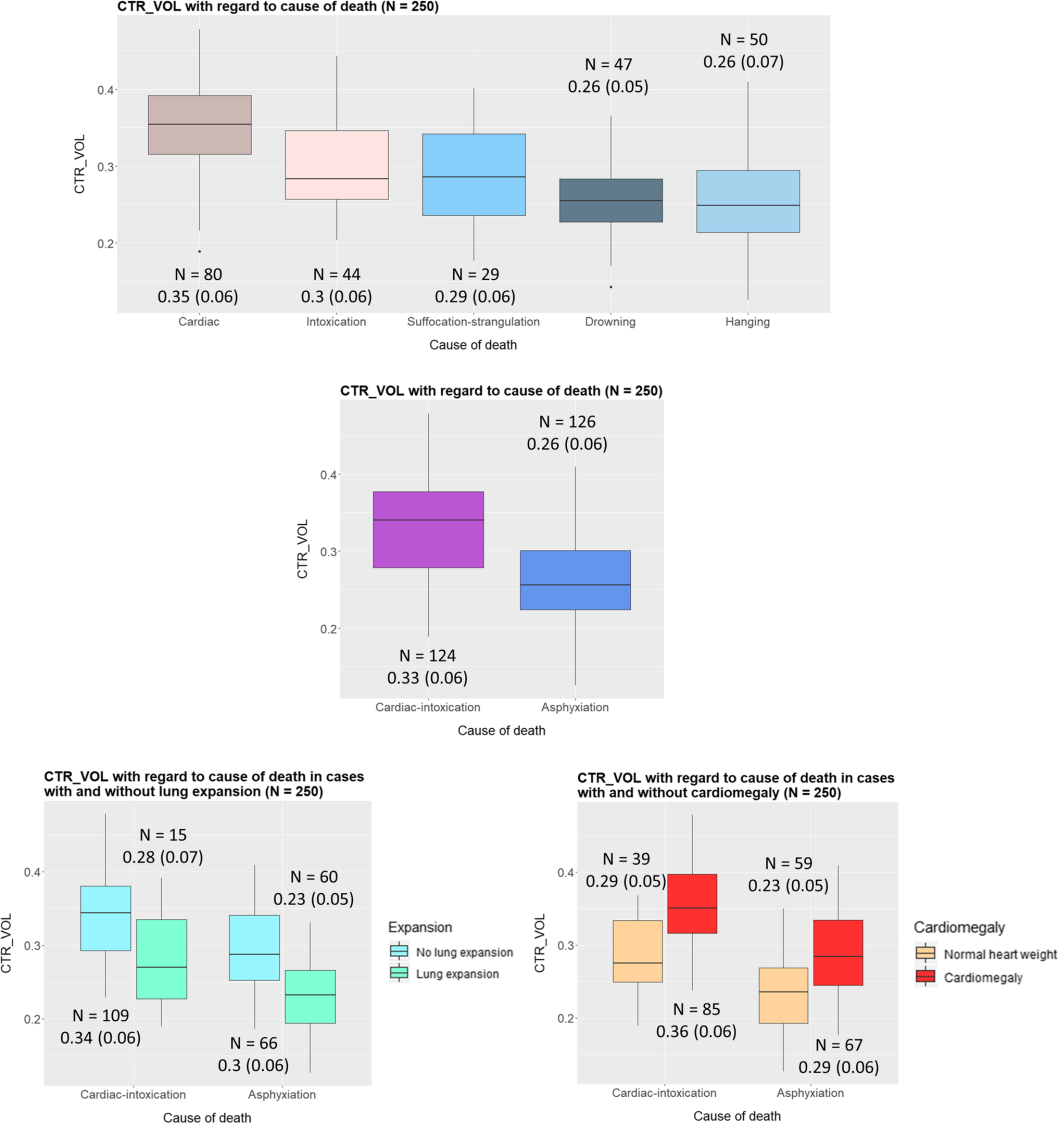
On the top: Boxplots depicting CTR_VOL in the different cause of death groups. Differences were found between cardiac deaths and all other causes of death, between intoxication and drowning, and between intoxication and hanging (pairwise post hoc: all *p* < 0.05). In the middle: CTR_VOL in the two cause of death categories independently of lung expansion or cardiomegaly. CTR_VOL was lower for “asphyxiation” compared to “cardiac-intoxication” (Student’s *t*-test: *p* < 0.001). Left on the bottom: CTR_VOL in the two cause of death categories with regard to lung expansion. CTR_VOL was lower for “asphyxiation” compared to “cardiac-intoxication” within the both lung expansion groups (within both expansion groups: Student’s *t*-test: *p* < 0.001). Right on the bottom: CTR_VOL in the two cause of death categories with regard to cardiomegaly. CTR_VOL was lower for “asphyxiation” compared to “cardiac-intoxication” within the distinct heart groups (within both heart groups: Student’s *t*-test: *p* < 0.001)

**Fig. 6:**
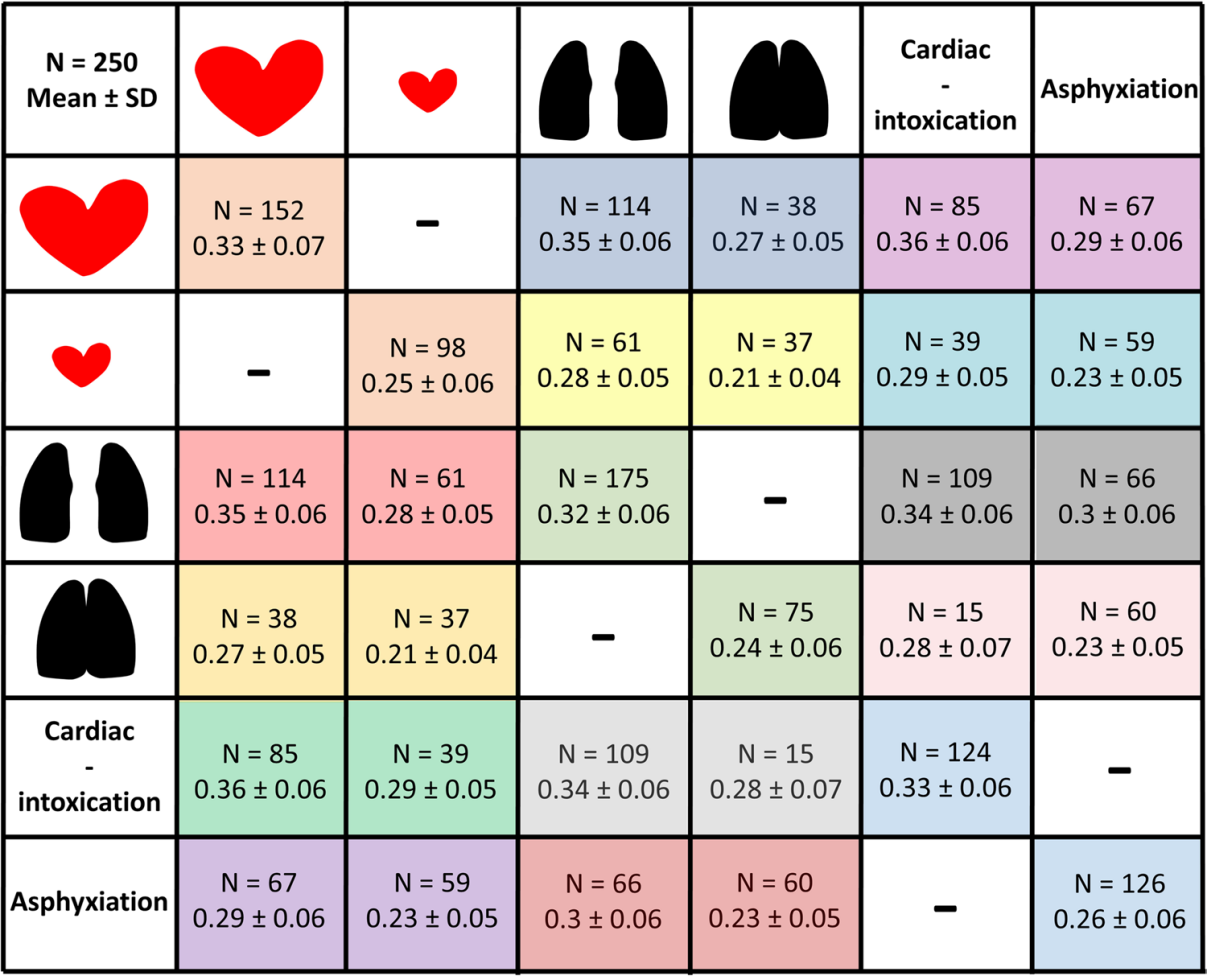
CTR_VOL variations with regard to cause of death category (“cardiac-intoxication” or “asphyxiation”), presence of cardiomegaly, and lung expansion—same-colored cells indicate statistically significant differences of CTR_VOL between the groups, e.g., yellow-colored cells: CTR_VOL was significantly larger in the group with normal weighted hearts and no lung expansion (*N* = 61, 0.28 ± 0.05) compared to the group with normal weighted hearts and presence of lung expansion (*N* = 37, 0.21 ± 0.04). CTR_VOL was significantly larger in “cardiac-intoxication” compared to “asphyxiation,” in cardiomegaly compared to normal weighted hearts and in no lung expansion compared to presence of lung expansion, respectively. CTR_VOL was larger for cardiomegaly compared to normal hearts and larger for “cardiac-intoxication” compared to “asphyxiation” for both present and absent lung expansion. CTR_VOL was smaller for lung expansion compared to no lung expansion and larger for “cardiac-intoxication” compared to “asphyxiation” in both cardiomegaly and normal hearts. CTR_VOL was larger for cardiomegaly compared to normal hearts and smaller for lung expansion compared to no lung expansion in both “cardiac-intoxication” and “asphyxiation.”

CTR_VOL for whole sample, for the distinct causes of death, heart, and lung expansion groups, is presented in Figs. 4, 5, and 6 and Table 2 and 3 in Supplementary material. CTR_VOL was larger for cardiomegaly compared to normal hearts and lower for lung expansion compared to no lung expansion (Figs. 4 and 6). There were differences of CTR_VOL among the distinct causes of death (Kruskal–Wallis: p < 0.001). Pairwise post hoc analysis with Dunn’s test showed larger CTR_VOL in cardiac deaths (Md = 0.35) than in all other causes (intoxication: Md = 0.28, suffocation-strangulation: Md = 0.28, drowning: Md = 0.26, hanging: M = 0.25, all *p*-values < 0.05). CTR_VOL in intoxication was significantly larger than drowning and hanging (both *p* < 0.05) (Fig. 5). Regarding cause of death categories, CTR_VOL was lower for “asphyxiation” compared to “cardiac-intoxication” in general (Student’s *t*-test: *p* < 0.001) (Fig. 5), as well as within the distinct lung expansion (within both expansion groups: Student’s *t*-test: *p* < 0.001) and heart groups (within both heart groups: Student’s *t*-test: *p* < 0.001) (Fig. 5, Fig. 6).

Univariately, it was shown that CTR_VOL% was positively associated with age (0.1% increase of CTR_VOL% by 1 year increase of age), with cardiomegaly (7% increase of CTR_VOL% in cardiomegaly under the assumption that the normal heart occupies approximately 0.2 × 100 = 20% of the thorax) and BMI (0.2% increase of the CTR_VOL% by every unit increase of BMI). CTR_VOL% was negatively associated with lung expansion (decrease of 8% in lung expansion) and asphyxiation (decrease of 7% in deaths related to asphyxiation). Gender was not associated with CTR_VOL%. In multivariable analysis, the same above-mentioned variables were still independently associated with CTR_VOL% (Table 2).

**Table 2:** Results of the univariate linear analysis and the multiple linear regression model

	Univariate analysis			Multivariable analysis*		
	Unadjusted *β*	95% CI	*p*-value	Adjusted *β*	95% CI	*p*-value
Age (years)	0.0015	(0.0010, 0.0019)	< 0.001	0.001	(0.0007, 0.0013)	< 0.001
Gender (male/female)	0.01	(− 0.009, 0.0288)	0.3	-	-	-
BMI (kg/m^2^)	0.0066	(0.0052, 0.0079)	< 0.001	0.0025	(0.0014, 0.0038)	< 0.001
Cause of death category (asphyxiation/cardiac-intoxication)	− 0.0701	(− 0.085, − 0.0537)	0.004	− 0.0333	(− 0.046, − 0.021)	< 0.001
Cardiomegaly at autopsy (cardiomegaly/normal heart weight)	0.0723	(0.056, 0.0884)	< 0.002	0.0287	(0.014, 0.042)	< 0.001
Lung expansion on PMCT (yes/no)	− 0.0848	(− 0.101, − 0.0682)	< 0.002	− 0.0588	(− 0.072, − 0.045)	< 0.001

*β* coefficient of the explanatory variable, *CI* confidence interval

^*^R^2^ = 0.62. Multiple regression formula:

CTR_VOL = 0.2 + 0.001 × age + 0.0025 × BMI + 0.0287 × cardiomegaly – 0.0588 × lung expansion – 0.0333 × cause of death category,

where cardiomegaly = 0 for normal heart weight and = 1 for cardiomegaly according to Zeek [14], lung expansion = 0 for no expansion and = 1 for present expansion, cause of death category = 0 for cardiac causes and intoxication and = 1 for asphyxiation-related deaths

Intrarater agreement was excellent for all three calculated volumes (right lung: ICC = 0.997, 95% CI: 0.996–0.998, *p* < 0.001; left lung: ICC = 0.998, 95% CI: 0.997–0.998, *p* < 0.001; mediastinum: ICC: 0.974, 95% CI: 0.966–0.979, *p* < 0.001).

## Discussion

Imaging comprises the best tool to examine the intact organs and assess positional relationships in situ [5]. CTR_VOL, considered a more accurate version of CTR, was assessed on PMCT to evaluate the relationship between postmortem heart and lungs in situ. This is the first attempt based on such a large sample by taking into account several influencing factors. The relatively high *R*^2^ value of the multivariate analysis indicates these factors have described a high percentage of the sample. Mediastinum occupies approximately 20% of the postmortem thoracic cavity in adults with normal BMI, normal-sized heart, and normally inflated lungs.

In a recent study, it was shown CTR on PMCT cannot reliably discriminate cardiomegaly alone and age, gender, and BMI should be taken into account [4]. Age showed significant effect in both analyses. However, CTR_VOL% increasing 0.1% for every year of age indicates a rather low effect in practice. In contrast, Zeek et al. did not observe any effect of age on heart weight [14]. Jotterand et al. did not find any association between age and CTR on PMCT in a sample with normal weighted hearts [3]. This association, however, was substantial for overweighed hearts [4], while taking into account gender and BMI for the determination of cardiomegaly [4]. Michiue et al. [5] found a mild correlation between CTR on postmortem chest X-rays and age.

Gender did not show any effect on CTR_VOL. This agrees with Jotterand et al. [3] and Michiue et al. [5], who also did not find any relationship between CTR on PMCT and gender [3, 5]. Zeek, however, supported that gender has an effect on human heart weight [14]. Despite males may have heavier hearts because of larger body build, the heart to thorax ratio may remain the same to that of women as males present larger rib cage and lung construction [24–26].

The approximately 0.3% increase of CTR_VOL% for every BMI-unit (β coefficient 0.0025) agrees with Zeek [14], who stated body length and nourishment affect heart weight [14] and Jotterand et al. [3], who found a correlation between CTR on PMCT and BMI for normal weighted hearts with a *β* coefficient 0.0022 [3], which is very close to the one observed in this current study.

Cardiomegaly was associated with higher CTR_VOL compared to normal hearts independently, as well as within all distinct groups. This is interesting indicating that present cardiomegaly cannot be overlaid in situ by any variation of the terminal cardiopulmonary pathophysiology. Cardiomegaly caused a 7% CTR_VOL% in the univariate model and 3% increase adjusted for all co-factors. Previous study, however, showed there is correlation between CTR and heart weight at autopsy only in cases died from heart diseases but not in cases died from asphyxiation and drowning, indicating lung hyperinflation may “mask” cardiomegaly on chest radiographs [5].

CTR_VOL in overweighed (M = 0.33) and normal hearts (M = 0.25) reflected the differences previously found for CTR (Michiue et al. [5]: CTR% on postmortem radiographs = 55.6%, Winklhofer et al. [2]: CTR on PMCT = 0.513 ± 0.07, Okuma et al. [27]: CTR on PMCT = 0.55 ± 0.08, Jotterand et al. [3, 4]: CTR on PMCT in normal hearts = 0.47 ± 0.06, in overweighed hearts = 0.53 ± 0.05). CTR_VOL values were similar to the 3D heart to lung ratio measured by Sogawa et al. [8] (heart/lung volume ratio ≈ 0.2–0.4, depending on cause of death); however, cardiomegaly was not assessed in this previous study. The heart to lung volume ratio was calculated [8], whereas the ratio of the mediastinal (and not only of the heart) to the whole thoracic volume (and not only the lungs) was calculated in the current study. Despite different measurement methods, 3D-CTR values by Sogawa et al. [8] were higher for sudden cardiac deaths (0.4) and intoxication (0.3) compared to drowning and mechanical asphyxia (both around 0.24). This is similar to those currently found with cardiac deaths (0.35) and intoxication (0.28) presenting higher CTR_VOL than suffocation-strangulation (0.28), drowning (0.26), and hanging (0.25). Sogawa et al. [8] described high variability within intoxication, which was also observed for intoxication with wider SD values in the current study.

Similar results demonstrated by Michiue et al. [5] with CTR on chest X-rays being higher for cardiac diseases/intoxication compared to drowning/asphyxiation. The first are associated with terminal organ congestion and lower aerated lung volumes (≈ 500 ml) and the second with lung hyperinflation and larger aerated lung volumes (≈1500 ml) [6]. Low CTR_VOL was expected for drowning because diaphragm levels are lower [5] and lungs appear emphysematous on PMCT compared to non-drowning [28, 29]. Christe et al. [28] compared CTR and the anterior distance between lungs on PMCT of 10 drowning compared to 20 non-drowning deaths and did not find any differences. The smaller sample [28] compared to our study may have led to different conclusions.

Lung expansion was evaluated separately and not only indirectly through the causes of death. It was assumed that terminal pulmonary mechanisms can individually vary and do not only strictly depend on cause of death itself. However, the sign of lungs touching each other anteriorly comprises just an indicator and does not describe hyperinflation in all of its aspects. Thus, cases with severe cardiac congestion or other mediastinal entities that mechanically impeded the lungs to expand till they reach each other anteriorly may have been assessed as without lung expansion in this study, though a hyperinflation aspect has been occurred and this comprises a limitation. Still, this sign seemed to play a crucial role in the positional relationship between lungs and heart. Mediastinum is compressed by the hyperinflated lungs causing approximately 8% CTR_VOL% decrease in the uni- and 6% in the multivariate analysis. Indications about this were described by Michiue et al. [5], who denoted low CTR in three cases with pulmonary emphysema.

One person conducted the manual segmentation and this is a limitation. However, CTR measurements and volumetric organ measurements have shown high interrater agreements [2, 3, 6, 30, 31]. Secondly, not all possible causes of death were assessed and hypothermia group was very small so the two hypothermia cases which fulfilled the inclusion criteria had to be excluded from the analysis. Presumably the low incidence of hypothermia deaths and the longer PMIs associated with such cases led to high rates of exclusion during the autopsy reports’ review. Thirdly, not all factors having a possible influence on CTR_VOL were assessed, especially cardiac dilatation [3, 6]. Fourthly, lung expansion was assessed based on a rather limited single sign. Fifth, this was a single-center study and influence of race could not be assessed. Last, mediastinal volume included paracardial fat, which may have led to CTR_VOL overestimation in cases with prominent paracardial fat deposits.

## Conclusion

Calculation of CTR_VOL is a viable technique to assess the relationship between the heart and lungs in situ. Age, BMI, and cardiomegaly increase, whereas asphyxiation-related causes of death and lung hyperinflation decrease CTR_VOL.

## Supplementary Information

Below is the link to the electronic supplementary material.

Supplementary file1 (DOCX 275 KB)

## Abbreviations

3D: Three-dimensional
CI: Confidence interval
CTR: Cardiothoracic ratio
CTR_VOL: Mediastinal-thoracic volume ratio
ICC: Intraclass correlation coefficient
M: Mean
Md: Median
PMI: Postmortem interval
PMCT: Postmortem computed tomography
