# Supplementary material for: Evaluation of the mediastinal-thoracic volume ratio on postmortem computed tomography
Source: Int J Legal Med. 2021 Apr 28;135(5):1903–12. doi: 10.1007/s00414-021-02593-0 (PMC8354949; doi:10.1007/s00414-021-02593-0)
Supplement: Supplementary file 1 — Supplementary file1 (DOCX 275 KB) [file 414_2021_2593_MOESM1_ESM.docx]

**Supplementary material**

**Pilot study in 3D Slicer software**

**Table 1 - appendix** Pilot study: the organ volumes and the mediastinal-thoracic ratio (CTR_VOL) extracted by down-sampled (10 mm slice thickness) and high resolution (5 mm thickness) PMCT-data did not significantly differ from each other confirming the method proposed by Ebert et al. [22].

|  | **Right lung volume (ml)** | | **Left lung volume (ml)** | | **Mediastinum volume (ml)** | | **CTR_VOL** | |
| --- | --- | --- | --- | --- | --- | --- | --- | --- |
| **Case** | 5 mm | 10 mm | 5 mm | 10 mm | 5 mm | 10 mm | 5 mm | 10 mm |
| **1** | 1671.57 | 1641.68 | 1392.77 | 1387.12 | 527.394 | 520.503 | 0.147 | 0.147 |
| **2** | 1158.95 | 1169.91 | 1054.02 | 1059.8 | 727.787 | 725.838 | 0.247 | 0.246 |
| **3** | 2428.95 | 2369.67 | 2101.44 | 2104.07 | 1426.32 | 1507.71 | 0.244 | 0.252 |
| **4** | 1423.31 | 1434.78 | 991.071 | 979.08 | 1360.34 | 1441.79 | 0.36 | 0.374 |
| **5** | 2396.97 | 2412.08 | 2153.44 | 2203.71 | 997.678 | 1003.79 | 0.18 | 0.179 |
| **6** | 1769.78 | 1793.36 | 1776.24 | 1769.9 | 1256.82 | 1236.62 | 0.262 | 0.258 |
| **7** | 1562.06 | 1567.69 | 1361.12 | 1350.35 | 986.017 | 991.29 | 0.252 | 0.254 |
| **8** | 1555.17 | 1533.76 | 1255.59 | 1265.3 | 2029.71 | 2017.31 | 0.419 | 0.419 |
| **9** | 1417.75 | 1405.48 | 1297.9 | 1301.79 | 766.438 | 746.532 | 0.22 | 0.216 |
| **10** | 1387.11 | 1380.71 | 927.205 | 899.382 | 1431.42 | 1443.03 | 0.382 | 0.388 |
| **Total**  **(M, SD)** | 1677.16,  422.22 | 1670.91,  414.24 | 1431.08,  439.05 | 1432.05,  452.42 | 1154.59,  443.37 | 1163.44,  452.45 | 0.271,  0.088 | 0.273,  0.091 |
| **p (t-test)** | 0.4 | | 0.8 | | 0.4 | | 0.4 | |

**Multiple regression model analysis**

Diagnostic plots were created for the multiple regression model (7) and indicated a well-functioning model (**Figure**). Backward elimination method was applied and showed the lowest Akaike Information Criterion (AIC) value for the initial model before starting deleting predictors indicating that no variable has to be excluded from the final model. R^2^ value was 0.62 indicating that this model can describe the 62% of the sample. The values of corrected GVIF were smaller than 41/1=4 for all predictors (age: 1.24, BMI: 1.42, lung expansion: 1.24, cardiomegaly: 1.54, cause of death: 1.21), thus there is not significant multicollinearity.


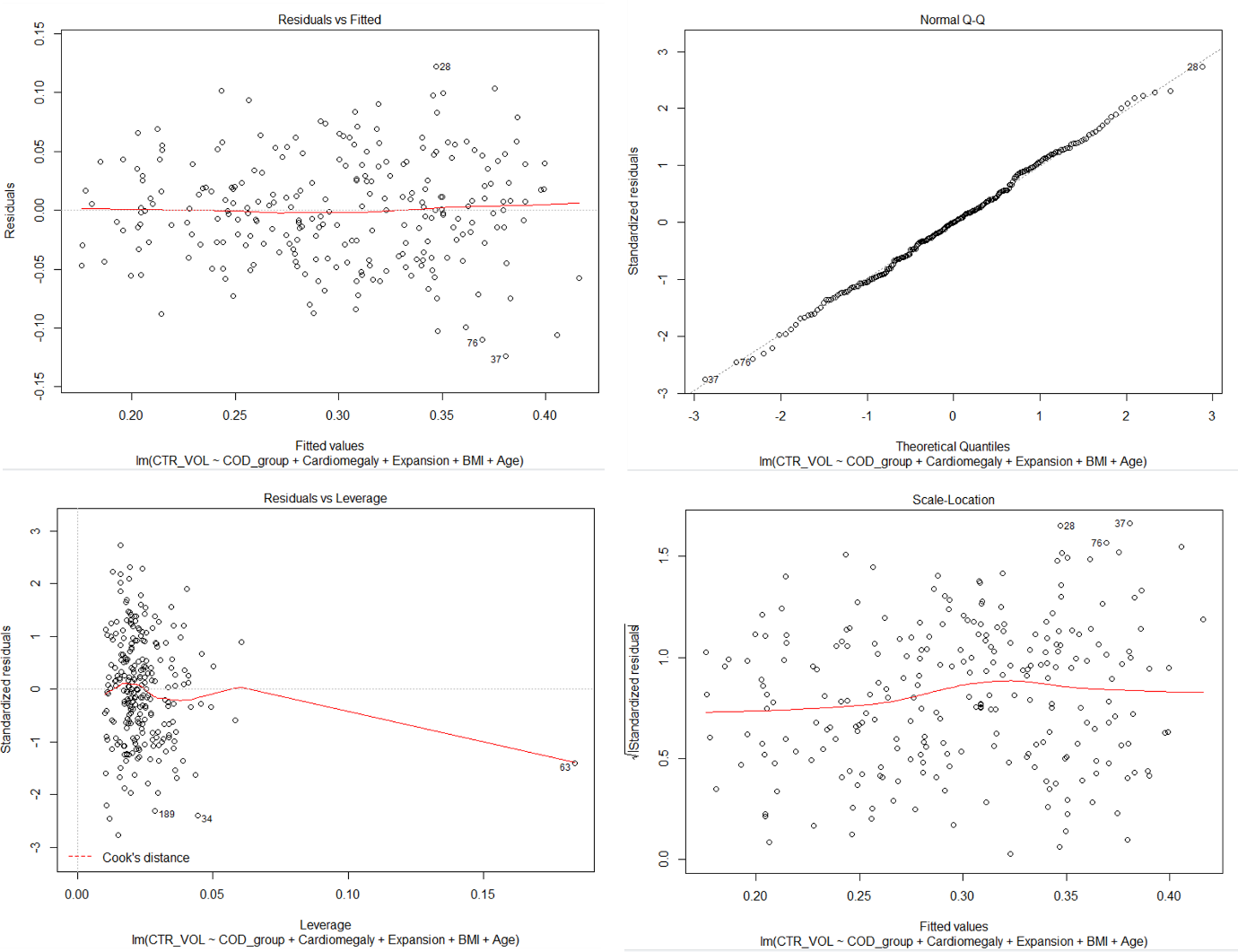


**Figure 1 – appendix** Diagnostic plots of the final model (equation (7)). Residuals vs fitted/predicted plot (left on the top): the residuals are equally spread around the line without distinct patterns indicating the existence of linear relationships. Normal Q-Q plot (right on the top): probability plot of the standardized residuals against the values that would be expected under normality. The residuals follow the 45-degree straight line and no prominent deviations are observed indicating normally distributed residuals. Residuals vs Leverage plot (left at the bottom): no observation shows a Cook’s distance > 1. Scale-Location graph (right at the bottom): the points are distributed randomly around the line indicating fulfilling of the constant variance assumption (homoscedasticity).

| **Table 2** Descriptive table depicting the CTR_VOL values within the cause of death groups. | | |
| --- | --- | --- |
| **CAUSE OF DEATH** | **N** | **CTR_VOL, Mean (SD)** |
| Cardiac | 80 | 0.35 (0.06) |
| Intoxication | 44 | 0.3 (0.06) |
| ***Category 1*** | 124 | 0.33 (0.06) |
|  |  |  |
| Suffocation-strangulation | 29 | 0.29 (0.06) |
| Drowning | 47 | 0.26 (0.05) |
| Hanging | 50 | 0.26 (0.07) |
| ***Category 2*** | 126 | 0.26 (0.06) |

| **Table 3** Mean CTR_VOL (SD) within the distinct lung expansion, cardiomegaly and cause of death groups. | | | | |
| --- | --- | --- | --- | --- |
| **CAUSE OF DEATH** | **Lung expansion –** (N= 177) | | **Lung expansion +** (N= 75) | |
| Cardiac (N= 80) | 0.36 (0.06) | | 0.29 (0.07) | |
| Intoxication (N= 44) | 0.31 (0.06) | | 0.27 (0.06) | |
| *Category 1 (N= 124)* | 0.34 (0.06) | | 0.28 (0.07) | |
| Suffocation-strangulation (N= 29) | 0.31 (0.06) | | 0.24 (0.05) | |
| Drowning (N= 47) | 0.27 (0.05) | | 0.23 ( 0.04) | |
| Hanging (N= 50) | 0.31 ( 0.06) | | 0.23 (0.05) | |
| *Category 2 (N= 126)* | 0.28 (0.06) | | 0.23 (0.05) | |
| **CAUSE OF DEATH** | **Cardiomegaly –** (N= 98) | | **Cardiomegaly +** (N= 152) | |
| Cardiac (N= 80) | 0.3 (0.04) | | 0.37 (0.07) | |
| Intoxication (N= 44) | 0.27 (0.05) | | 0.33 (0.05) | |
| *Category 1 (N= 124)* | 0.29 (0.05) | | 0.36 (0.06) | |
| Suffocation-strangulation (N= 29) | 0.26 (0.05) | | 0.32 (0.07) | |
| Drowning (N= 47) | 0.23 (0.05) | | 0.27 (0.05) | |
| Hanging (N= 50) | 0.22 (0.05) | | 0.29 (0.06) | |
| *Category 2 (N= 126)* | 0.23 (0.05) | | 0.29 (0.06) | |
|  | **Cardiomegaly –** (N= 98) | | **Cardiomegaly +** (N= 152) | |
| **CAUSE OF DEATH** | **Lung expansion –**  (N= 61) | **Lung expansion +**  (N= 37) | **Lung expansion –**  (N= 114) | **Lung expansion +**  (N= 38) |
| Cardiac (N= 80) | 0.32 (0.04) | 0.23 (0.04) | 0.37 (0.06) | 0.33 (0.06) |
| Intoxication (N= 44) | 0.28 (0.04) | 0.22 (0.01) | 0.33( 0.05) | 0.3 (0.06) |
| *Category 1 (N= 124)* | 0.3 (0.05) | 0.22 (0.03) | 0.36 (0.06) | 0.32 (0.06) |
| Suffocation-strangulation (N= 29) | 0.29 (0.06) | 0.22 (0.04) | 0.33 (0.06) | 0.27 (0.08) |
| Drowning (N= 47) | 0.25 (0.04) | 0.19 (0.04) | 0.29 (0.05) | 0.25 (0.02) |
| Hanging (N= 50) | 0.26 (0.04) | 0.2 (0.05) | 0.34 (0.06) | 0.26 (0.04) |
| *Category 2 (N= 126)* | 0.27 (0.04) | 0.2 (0.04) | 0.32 (0.06) | 0.25 (0.04) |
